# Long term complications and prognostic factors in locally advanced nasopharyngeal carcinoma treated with docetaxel, cisplatin, 5-fluorouracil induction chemotherapy followed by concurrent chemoradiotherapy: A retrospective cohort study

**DOI:** 10.1097/MD.0000000000023173

**Published:** 2020-12-04

**Authors:** Hyun Jeong Shim, Hyeon Jong Kim, Jun Eul Hwang, Woo Kyun Bae, Ik Joo Chung, Dong Hoon Lee, Yoon Tae Mi, Joon Kyoo Lee, Sang Chul Lim, Jae Wook Chung, Sang Hee Cho

**Affiliations:** a Department of Hematology and Oncology; b Otorhinolaryngology-Head and Neck Surgery; c Radiation Oncology, Chonnam National University Medical School, Gwangju, Korea.

**Keywords:** chemoradiotherapy, complications, induction chemotherapy, nasopharyngeal carcinoma

## Abstract

This study was conducted to evaluate the long term complications and their risk factors including of survival outcomes in patients with locally advanced nasopharyngeal cancer (NPC) treated with docetaxel, cisplatin and 5-fluorouracil (TPF) induction chemotherapy followed by concurrent chemoradiotherapy (CCRT).

Among the patients who were diagnosed as NPC, we consecutively evaluated the late complications in 104 patients who completed 3 cycles of TPF induction chemotherapy followed by CCRT and received regular follow-up by otolaryngologist and oncologist. The prognostic factors for overall survival, relapse free survival and each complication were analyzed based on clinical characteristics.

Over a median follow-up of 54 months (range, 7.9–152.9 months), 5-year overall survival rate was 87% for stage II, 89% for stage III, 87% for stage IV patients. The significant prognostic factor for survival is complete response rate after CCRT in multivariate analysis. The most frequent toxicity was ear complication (29.8%) including of hearing loss requiring hearing aid (6.7%) and bone necrosis (3.8%). Decreased renal function over grade 2 was occurred in only 4 patients (3.8%) regardless of the cumulative dose of cisplatin. The long term complications did not affect the survival outcome. Patients who received radiation therapy more than 5400 cGy had better survival outcome than those who did not. However, ear complication was significantly related to radiation dose (≥ 6,600 cGy) and type of radiation therapy (conventional). Age over 65 years was a significant risk factor for both ear and renal toxicity. In conclusion, close follow-up to monitor long-term complications should be performed in patients treated with TPF induction chemotherapy followed by CCRT treatment, especially in elderly patients. Reestablishing the optimal chemotherapeutic agent during CCRT and adjustment of radiation dose after induction chemotherapy could be helpful to reduce the toxicity associated with the subsequent treatment strategy for locally advance NPC patients.

## 1 Introduction

A multidisciplinary treatment approach has been widely performed in various cancer. In particular, head neck cancer is actively being treated using combined modality including of surgery, radiation therapy (RT), and chemotherapy. Among head and neck cancers, nasopharyngeal cancer (NPC) is distinguished from other epithelial head and neck cancers in several points. First, its geographically endemic distribution suggests that a combination of genetic, ethnic, and environmental factors, including EBV infection, affects the pathogenesis of NPC. These findings are different from other head and neck cancers, which are commonly associated with smoking or alcohol. In addition, NPC typically develops in the deeper part of the head and easily invades into the surrounding tissues; thus, surgery is not easily considered as the first line of therapeutic option. Fortunately, given its high radiosensitivity, RT could be the standard treatment for NPC with or without concurrent chemotherapy for non-metastatic disease. As the development of the RT method, intensity modulated RT (IMRT) are widely used rather than conventional RT, and thus, a sufficient dose of radiation to kill tumor cell is administered with reduced toxicity. Also, the use of concurrent chemoradiotherapy (CCRT) to enhance the treatment outcome has extended patient survival compared with RT alone.^[1–4]^ However, the incidence of local or distant recurrence still remains unsatisfactory despite these efforts. Thus, additional treatments for RT or CCRT are being explored, and 1 of those attempts is to introduce additional chemotherapy before (induction) or after (adjuvant) CCRT.

In a recent prospective study using adjuvant chemotherapy with gemcitabine and cisplatin after RT for high-risk patients based on the plasma EBV DNA level, the 5-year relapse-free survival (RFS) and overall survival (OS) did not improve.^[5,6]^ The possible reasons for this disappointing result may be associated with the poor tolerance to chemotherapy after RT completion (50%–76% compliance) and mitigation of the chemotherapy due to radiation-induced tissue damage.^[1,2,7]^ The induction chemotherapy followed by RT or CCRT in locally advanced NPC has been evaluated extensively, and its survival benefit has been shown in several meta-analyses.^[1,3,4]^ Compared with adjuvant chemotherapy, induction chemotherapy is better tolerable since it is administered before RT and can reduce micrometastasis earlier because higher doses of chemotherapy are used than those during RT.^[8–10]^ In addition, a complete response (CR) after CCRT is considered as an important prognostic factor for survival, and induction chemotherapy may increase CR rate through shrinkage of bulky mass before CCRT. Considering these advantages, induction chemotherapy has been performed for some selected patients with locally advanced NPC.

Previously, we reported the efficacy and safety of induction in bulky chemotherapy consisting of docetaxel, cisplatin, and 5-fluorouracil (TPF) followed by CCRT for patients with locally advanced NPC, and the survival benefit of this treatment modality has been demonstrated in subsequent phase III trials.^[11,12]^ Nevertheless, the benefits observed were inconsistent, possibly due to insufficient patient enrollment, different induction regimens or chemotherapeutic agents during RT. In addition, recent study using gemcitabine and cisplatin as an induction regimen showed promising result with reduced toxicity. Thus, the role of TPF regimen may become less highlighted. However, TPF regiment shows clear evidence of the effectiveness, especially in EBV positive NPC and other agents such as gemcitabine may be difficult to select due to problem such as reimbursement. Therefore, the TPF regimen is still used as a treatment option in locally advanced NPC.

Not only prolongation of survival but also improving quality of life is important, the evaluation of toxicity of TPF followed by CCRT should be re-evaluated. The acute toxicities caused by this sequential treatment are well known; however, little is known about long term complications and their risk factors. In addition, the optimal dose of radiation or cisplatin during RT after induction chemotherapy has not been established, which may be associated with toxicity.

Therefore, this study was conducted to retrospectively evaluate the long term complications with their risk factors and survival outcomes. Based on these findings, we aimed to suggest the guideline of regular follow for high risk patients and the optimal dose of radiation or cisplatin during radiation considering both therapeutic effect and toxicity.

## 2 Methods

### 2.1 Patients

**Figure 1:**
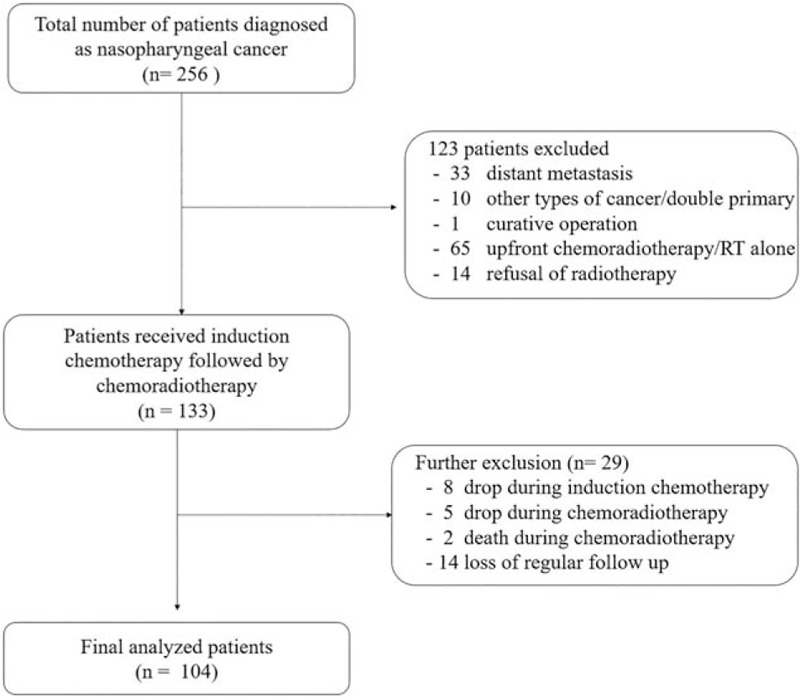
Consort diagram.

From May 2005 to January 2015, 256 patients were diagnosed with NPC at Chonnam National University Hwasun Hospital. Of these, 133 patients were planned to receive the induction chemotherapy followed by CCRT based on staging workup. But, 15 patients discontinued this treatment due to toxicities and 14 patients did not undergo regular follow up. Therefore, this study was analyzed in 104 patients retrospectively, as per protocol study, who received 3 cycles of induction TPF followed by CCRT. The study flow is shown in the CONSORT diagram (Fig. 1) and data analysis was performed until Dec 31, 2019.

To evaluate the long term complication, this study was analyzed in patients who completed 3 cycles of TPF induction chemotherapy and CCRT and underwent regular follow up to monitor disease recurrence and complications by an otolaryngologist and medical oncologist. Pretreatment staging involved examination of the ears, nose, and throat by an otolaryngologist, as well as computed tomography (CT) or magnetic resonance imaging evaluation of the primary tumor site and lymph node. The 8th edition of the American Joint Committee on Cancer staging system was used for staging. To detect other primary malignancies or metastases, patients underwent a CT scan of chest and abdomen as baseline. Before RT, routine dental examinations were performed to prevent unexpected osteonecrosis or osteomyelitis associated with RT. This study was approved by the Chonnam National University Hwasun Hospital Institutional Review Board (CNUHH-2019-155).

### 2.2 Chemotherapy schedule and RT

For induction chemotherapy, docetaxel (70 mg/m^2^) and cisplatin (75 mg/m^2^) were administered as 4-h intravenous infusions on day 1, followed by 5-fluorouracil (1,000 mg/m^2^) administered as a 24-h continuous infusion for 4 days. The cycles were repeated every 3- weeks, and a total of 3 cycles of induction chemotherapy were administered to the patients. RT was started 3 to 4 weeks after completion of the third cycle of induction chemotherapy. During RT, cisplatin was administered at a dose of 100 mg/m^2^ 3 times every 3 weeks (3-week regimen) or 40 mg/m^2^ weekly (weekly regimen) for 7 weeks depending on the patient's condition or physician's preference. Cisplatin was administered based on creatinine clearance (Clcr). RT was performed using 6 MV photon beams produced by a linear acceleration with 2-dimensional conformal RT (2D-CRT), 3D-CRT via a shrinking field technique using the Clinac IX (Varian Medical Systems, CA), or with IMRT, using the Clinac IX or TomoTherapy (Accuray, Sunnyvale, CA). Patients with gross disease remaining at the neck nodes or primary site were administered 66 to 70 Gy at 2 Gy per fraction 5 days a week. Elective nodal irradiation was administered at a dose of 45 to 50 Gy.

### 2.3 Follow-up evaluation of tumor response and long term complications

After 3 cycles of induction chemotherapy and at 8 to 10 weeks after completion of CCRT, the patient's clinical response was assessed by a physical examination conducted by an otolaryngologist, as well as by CT imaging of the primary tumor and neck. A biopsy of the primary site or lymph nodes was recommended if the remaining lesion was suspicious. The tumor response was assessed according to the Response Evaluation Criteria in Solid Tumors version 1.1. For all patients with a CR according to the physical examination and CT, an additional CT or magnetic resonance imaging scan was performed 1 month later if necessary to confirm the CR.

The patients were followed up to reassess their disease status and monitor toxicities after treatment completion; CT and toxicity evaluation were performed every 4 months for 2 years. Thereafter, physical examination and CT were performed every 6 months until disease progression. Patients with locoregional recurrence and/or metastatic disease had the options of receiving repeat RT, surgery, and/or palliative chemotherapy based on the patient's condition. The Common Toxicity Criteria for Adverse Events (CTCAE v4.0) system was used to evaluate toxicity, especially in ear and labyrinth disorder. The highest grade of toxicity was recorded for analysis in this study.

### 2.4 Statistical analysis

Association analysis with the incidence of long term complication and clinical parameters were performed using the chi-square test and Fisher exact test. The clinical outcomes were RFS and OS. The median OS was measured from the start of chemotherapy until the date of death or the last confirmed date of survival. The RFS was defined as the time from the start of chemotherapy to the first observation of relapsed disease or death from any cause. Survival curves (OS and RFS) were calculated using the Kaplan–Meier method and curves were compared using the log-rank test. The patients who are lost to follow-up at the data of analysis are censored. Univariate analysis was performed using Kaplan–Meier method for age, T stage, N stage, AJCC stage, tumor response, cumulative RT and cisplatin dose. The Cox proportional hazards model was used for multivariate analyses to calculate hazard ratios (HRs) for the association of independent factors with survival. SPSS version 21.0 (SPSS, Inc., Chicago, IL) was used for the statistical analyses. A 2-tailed *P* < .05 was considered statistically significant.

## 3 Results

### 3.1 Treatment outcomes

**Figure 2:**
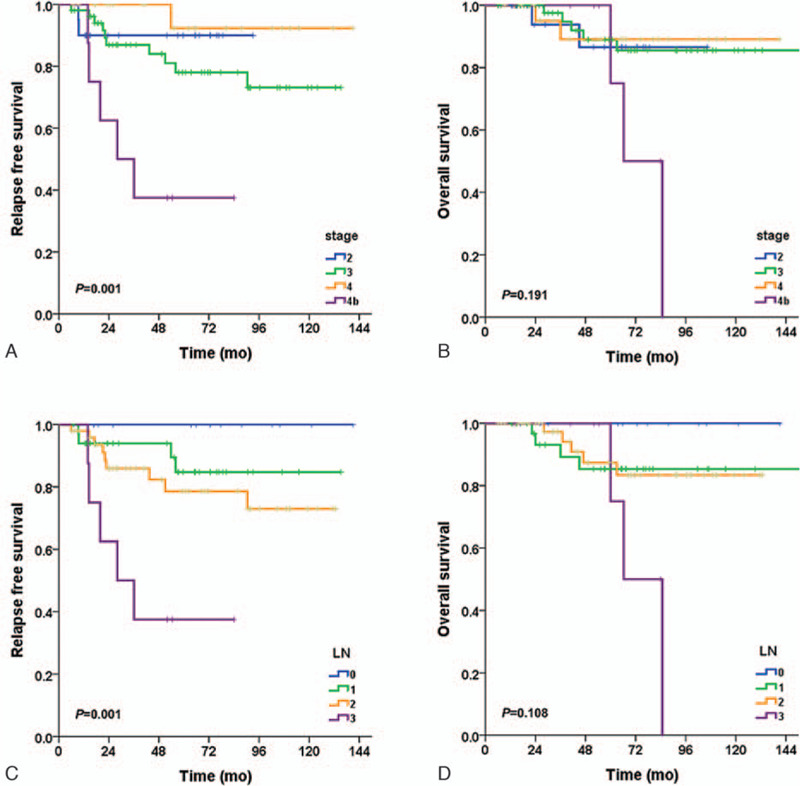
Kaplan-Meier relapse free survival and overall survival curves for TNM stage (A, B) and N stage (Fig C, D) in 104 patients with locally advanced nasopharyngeal cancer.

The baseline patient characteristics are shown in Table 1. After a median follow-up of 54 months (range, 26–141 months), the 3-year RFS and 5-year OS rates were 90% (95% confidence interval [CI] 77–103) and 87% (95% CI 69–104) for stage II patients, 87% (95% CI 77–97) and 89% (95% CI 79–99) for stage III patients, and 80% (95% CI 64–96) and 87% (95% CI 69–104) for stage IV patients, respectively (Fig. 2A, Fig. 2B).

**Table 1:** Patient and disease characteristics.

Characteristics	Number (%)
Total patients	104
Age (median, range)	52 (14–76)
≥65 yr	18 (17)
< 65 yr	86 (83)
Gender (N, %)	
Male	75 (72)
Female	29 (28)
ECOG PS	
0	82 (79)
1	17 (16)
2	5 (5)
EBV association	
Negative	5 (5)
Positive	84 (81)
Unknown	15 (14)
Tumor (T)	
T1	21 (20)
T2	39 (38)
T3	19 (18)
T4	25 (24)
Lymph node (N)	
N0	12 (11)
N1	34 (33)
N2	50 (48)
N3	8 (8)
AJCC (8^th^)	
II	20 (19)
III	53 (51)
IVA	23 (22)
IVB	8 (8)
During chemoradiotherapy	
median dose of cumulative cisplatin	175 mg/m^2^
median dose of cumulative radiation	6,600 cGy
Type or RT technique	
Conventional RT	79 (76%)
3D/IMRT	25 (24%)

IMRT = intensity modulated radiation therapy, PS = performance score, RT = radiation therapy.

After induction chemotherapy, a complete response (CR) was observed in 24 patients (23%; 8 with stage II, 13 with stage III, and 3 with stage IV), and these patients are still alive at the time of this analysis. As expected, the CR rate after induction chemotherapy was significantly higher in patients with a locally advanced stage (II or III) than in those with an advanced stage (IV, *p* = 0.027). After CCRT, 89 patients (86%) achieved a CR, and patients with a CR after induction chemotherapy (*P* = .044) or CCRT (*P* = .002) had a better OS regardless of stage (Table 2).

**Table 2:** Tumor response to induction chemotherapy and chemoradiation therapy.

Response	Primary lesion (N = 104)	Lymph node (N = 92)
After induction chemotherapy		
CR	56 (54)	42 (40)
PR	46 (44)	61 (59)
SD	2 (2)	1 (1)
PD	0	0
Overall response		
CR (N, %)	24 (23)	
non-CR (N, %)	80 (77)	
After completion of chemoradiotherapy		
CR (N, %)	89 (86)	
non-CR (N, %)	15 (14)	

CR = complete response, PD = progressive disease, PR = partial response, SD = stable disease.

During CCRT, the median dose of radiation was 6,600 cGy, and the completion rate of RT was 75% (n = 78). Seventy-nine patients (76%) and 25 patients (24%) received 3D/IMRT and conventional 2D-RT, respectively. During CCRT, 7 patients experienced an interruption in RT over 5 days, and 6 patients received a radiation dose less than 5,000 cGy. Cisplatin was administered during RT, with 47 patients (45%) receiving weekly regimen and 57 patients (55%) receiving 3-week regimen. The median dose of cisplatin was 175 mg/m^2^, and the cumulative dose of cisplatin was higher in patients with 3-week regimen (183.4 ± 58 mg/m^2^) than in patients with weekly regimen (155.7 ± 68 mg/m^2^, *p* = 0.024).

There was no difference in the cumulative radiation dose based on the age of 65, but the cumulative cisplatin dose was significantly higher in patients under 65 years (176.4 ± 64.8 mg) than those more than 65 years (144.4 ± 43.8, *P* = .049).

### 3.2 Prognostic factors for response and survival outcomes

Age and the T stage were not associated with the CR rate, RFS, or OS; however, the N stage was significantly associated with RFS and OS (Fig. 2C, Fig. 2D). N3 stage and a non-CR after CCRT were associated with poor survival outcome on univariate analyses, and a non-CR after CCRT was a significant prognostic factor for RFS and OS on the multivariate analysis (Table 3). Because no recurrence or death occurred among the patients with a CR after induction chemotherapy, the Cox proportional hazards model could not be applied for multivariate analyses about N stage and AJCC staging.

**Table 3:** Univariate and multivariate analysis for survival outcome.

	Univariate analysis				Multivariate analysis			
	RFS HR (95% CI)	*p*	OS HR (95% CI)	*p*	RFS HR (95% CI)	*p*	OS HR (95% CI)	*p*
Age (≥ 65 yr)	2.242 (0.793–6.342)	.128	1.492 (0.324–6.871)	.608	2.242 (0.793–6.342)	.128	1.492 (0.324–6.871)	.608
T stage (T3–4)	1.431 (0.820–2.495)	.207	0.809 (0.243–2.696)	.730	1.431 (0.820–2.495)	.207	0.809 (0.243–2.696)	.730
N stage (N3)	5.387 (1.874–15.484)	.002	3.739 (1.004–13.927)	.049	5.387 (1.874–15.484)	.002	3.739 (1.004–13.927)	.049
Stage, AJCC 8th (IV)	0.918 (0.562–1.499)	.732	1.566 (0.497–4.935)	.444	0.918 (0.562–1.499)	.732	1.566 (0.497–4.935)	.444
Response after CCRT (non-CR)	3.393 (1.272–9.053)	.015	4.942 (1.592–15.348)	.006	3.393 (1.272–9.053)	.015	4.942 (1.592–15.348)	.006
Cumulative RT (<6,600 cGy)	0.502 (0.194–1.295)	.154	0.732 (0.220–2.432)	.610	0.502 (0.194–1.295)	.154	0.732 (0.220–2.432)	.610
Cumulative cisplatin (<175mg/m^2^)	0.670 (0.259–1.733)	.409	1.115 (0.335–3.707)	.860	0.670 (0.259–1.733)	.409	1.115 (0.335–3.707)	.860

CCRT = concurrent chemoradiotherapy, OS = overall survival, RFS = relapse free survival, RT = radiation therapy.

The CR rate after CCRT was significantly higher in patients who received cisplatin weekly than in patients who received cisplatin receiving 3-week regimen (*P* = .030), although the cumulative dose of cisplatin was higher in patients receiving 3-week regimen than in patients with weekly regimen. The cumulative dose of cisplatin (≥175 mg/m^2^ vs <175 mg/m^2^) and radiation (≥6600 cGy vs <6,600 cGy) during RT, age (< 65 vs. ≥ 65 years) and the type of RT technique did not affect the CR rate or survival outcomes.

### 3.3 Incidence and pattern of relapse during follow-up

Recurrence was observed in 18 patients (17.3%). Ten patients experienced distant metastasis in the lung, liver, and bone, in that order of frequency. Nine patients experienced local recurrence (regional lymph node metastasis in 4 patients and primary recurrence in 5 patients), and 1 patient experienced local and distant metastasis. Five patients received surgical resection and 10 patients received palliative chemotherapy. At the time of analysis, eight patients were alive, and 3 patients had no evidence of disease after salvage surgery. The median survival after recurrence was 38 months (95% CI, 28.7–47.2). The patients with a short progression-free survival (<2 years) showed a significantly poorer OS compared with patients with a longer progression-free survival (>2 years; *P* = .017).

### 3.4 Long term complications and the associated risk factors

**Figure 3:**
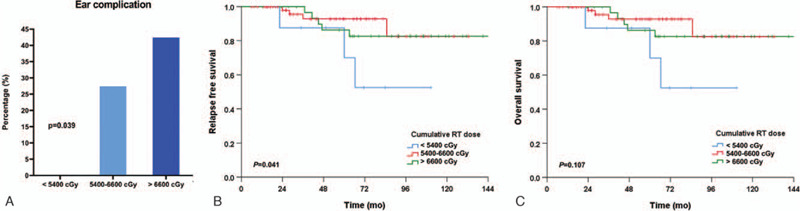
Ear complication and survival outcomes according to radiation dose.

The most frequent toxicity was ear complication (29.8%) including of hearing loss requiring hearing aid (6.7%) and bone necrosis (3.8%). Infections, such as chronic otitis and fungal infections, developed in 15 patients, hearing impairment in 9 patients, tinnitus or dizziness that required medication in 5 patients, ear drum perforation in 10 patients, and bone radionecrosis in 2 patients. As shown in Table 4, age was an important risk factor for both renal and ear complications. In addition to age, the radiation dose (≥6,600 cGy) and type of RT (conventional) were significantly associated with the incidence of ear complications. To estimate the radiation dose causing ear complication, we stratified the patients into 3 groups according to the cumulative radiation dose: < 5400 cGy (n = 9), 5400–6600 cGy (n = 62) and > 6600 cGy (n = 33). The incidence of ear complications increased significantly in proportion to cumulative radiation dose (*p* = 0.039). Next, we analyzed survival outcomes in each group according to the cumulative radiation dose to find the correlation between radiation dose and therapeutic effect. There was no difference in the CR rate, but RFS and OS were significantly extended in the group receiving above 5400 cGy compared to the group below. Interestingly, there was no difference in survival outcomes between patients receiving radiation dose of 5400–6600 cGy and those receiving radiation dose above 6600 cGy (Fig. 3). The cumulative dose of cisplatin during RT did not affect the incidence of ear complication.

**Table 4:** Long term complications after induction chemotherapy followed by chemoradiotherapy in nasopharyngeal cancer.

	Renal complication, N (%)^∗^			Ear complication, N (%)		
	-	+	*p*	-	+	*p*
Age			.044			.011
< 65 yr	65 (76)	21 (24)		65 (76)	21 (24)	
≥ 65 yr	9 (50)	9 (50)		8 (44)	10 (56)	
Baseline Clcr			.289			.424
≥ 60 mL/min/L.73m^2^	68 (73)	25 (23)		66 (71)	27 (29)	
< 60 mL/min/1.73m^2^	6 (55)	5 (45)		7 (64)	4 (36)	
Clcr before CCRT			0.003			.250
≥ 60 mL/min/1.73m^2^	64 (79)	17 (21)		60 (72)	23 (27)	
< 60 mL/min/1.73m^2^	9 (44)	13 (56)	0.491	13 (62)	8 (38)	.260
Use of cisplatin regimen						
3-weekly	40 (70)	17 (30)		42 (74)	15 (26)	
weekly	34 (72)	13 (28)		31 (66)	16 (34)	
Cumulative cisplatin dose^†^			0.536			.032
<175mg/m^2^	36 (71)	15 (29)		31 (61)	20 (39)	
≥175mg/m^2^	38 (72)	15 (28)		42 (79)	11 (21)	
Cumulative radiation dose			0.507			.050
< 6600 cGy	19 (73)	7 (27)		22 (85)	4 (15)	
≥6600 cGy	55 (71)	23 (29)		51 (65)	27 (35)	
Type of RT						.000
3D/IMRT				63 (80)	16 (20)	
2D RT				10 (40)	15 (60)	

CCRT = concurrent chemoradiotherapy, IMRT = intensity modulated radiation therapy, RT = radiation therapy.

∗ Renal complication is defined as < 60 mL/min/1.73m2 at last follow up data.

† Cumulative cisplatin dose is defined as the dose during radiotherapy.

Regarding renal toxicity, decreased renal function over grade 2 was occurred in only 4 patients (3.8%) regardless of the cumulative dose of cisplatin and there was no patient showing more than grade 3 adverse event. The decreased renal function with Clcr 60 mL/min/1.73m^2^ or less was observed in 30 patients, frequently in elderly (≥65 years) and in patients with a low Clcr before starting CCRT (Table 4).

## 4 Discussion

Although the role of induction chemotherapy for NPC treatment is controversy, significant improvements in survival outcomes were reported when induction chemotherapy was followed by CCRT in NPC: a 6% absolute increase in the OS rate and 7% absolute reduction in the distant failure rate at 5 years compared with CCRT alone.^[13]^ According to these results, the 2018 NCCN guidelines updated the evidence for induction chemotherapy with CCRT from level 3 to level 2A, which is equivalent to that for adjuvant chemotherapy with CCRT. In addition, in a recent phase III trial using induction chemotherapy consisting of gemcitabine and cisplatin, a significant improvement in survival was observed, thus emphasizing the importance of this sequential therapy for the management of NPC.^[14]^

Along with these survival results, acute complications have been reported extensively. However, long term complications and their risk factors caused by induction chemotherapy followed by CCRT have not been well investigated. Ear complication is more common than renal toxicity due to the use of radiation and nephrotoxic agents, such as cisplatin. Du et al reported that ear complications such as deafness/otitis, was the most common toxicity, occurring in 20.7% in the CCRT group and 13.3% in the RT alone group (RR = 1.567; 95% CI 1.192–2.152); however, the risks of other types of severe late toxicities were not significantly different between the groups.^[15]^ The addition of induction chemotherapy may further increase the toxicity. Therefore, it is important to analyze the actual incidences of late complications and the associated risk factors in practice. Recently, Yang et al reported that patients who received induction chemotherapy consisting of 2 cycles of 5-fluorouracil and cisplatin followed by CCRT showed long-term disease-free survival and OS benefits, without any differences in late complications compared with CCRT alone group.^[16]^

Regarding toxicities, the dose and interval of cisplatin administration are important issues. In cases of upfront CCRT for head and neck cancer, a high dose of cisplatin with 3 times of 100 mg/m^2^ every 3 weeks is recommended as a standard therapy. Generally, the cumulative cisplatin dose of 200 mg/m^2^ during RT is generally accepted as the minimum optimal dose to obtain a survival benefit.^[17]^ However, when induction chemotherapy is added prior to CCRT, the optimal cisplatin dose and delivery interval should be reconsidered. Lv et al showed that the cumulative cisplatin dose (>200 mg/m^2^ vs ≤200 mg/m^2^) during RT was not associated with survival outcomes in patients receiving induction chemotherapy plus CCRT, and that 160 mg/m^2^ cisplatin may be sufficient to yield beneficial antitumor effects.^[18]^ This result is similar to the 175 mg/m^2^ dose that we reported. However, Liu et al reported the importance of a cumulative cisplatin dose more than 200 mg/m^2^ after induction chemotherapy, showing higher 3-year progression-free survival rates in patients receiving cisplatin more than 200 mg/m^2^ compared with under 100mg/m^2^.^[19]^ In a phase III trial using TPF as induction chemotherapy, a significant difference in the cumulative cisplatin dose durint RT was observed according to the addition of induction chemotherapy; the mean relative dose intensity for concurrent cisplatin was 71% in the induction chemotherapy + CCRT group versus 84% in the CCRT alone group (*P* < .0001).^[12]^ In our results, we analyzed the response rate and survival outcomes in 3 groups of < 170 mg/m^2^ (n = 47), 170–200 mg/m^2^ (n = 32), > 200 mg/m^2^ (n = 25) according to the cumulative cisplatin dose. But there were no significant differences between the groups (data not shown). Unexpectedly, our study showed that higher incidences of ear complications were observed in the low-dose cisplatin group (< 175 mg/m^2^) than in the high-dose cisplatin group (>175 mg/m^2^). This may have been associated with age, because of the high proportion of elderly patients in the low-dose cisplatin group (*P* = .007), and ear complications are more common in elderly patients (*P* = .039). Thus, it is necessary to be careful interpretation for the results, and further study is needed to define the role of cisplatin in complication and treatment outcome.

Based on previous and our results, we can propose optimal cumulative cisplatin dose as 160–200 mg/m^2^ during CCRT after induction chemotherapy. Recently, nedaplatin, which was developed to decrease toxicities, has been used instead of cisplatin. Tang et al reported that auditory/hearing adverse events greater than grade 1 occurred significantly less frequently in the nedaplatin group than in the cisplatin group (*P* = .0014) during CCRT for locally advanced NPC.^[20]^ Thus, nedaplatin can be used as an alternative agent during RT after induction chemotherapy to avoid toxicities.

Another issue regarding cisplatin is the treatment interval. A higher dose of cumulative cisplatin was delivered by the 3-week regimen than by the weekly regimen in the present study. However, the risks of late ototoxicity and treatment interruption during RT were higher in the 3-week regimen, and RT interruption may cause a decreased local control rate.^[15,21]^ Conversely, the weekly cisplatin regimen is more tolerable, and the maximal concentration of cisplatin is relatively low compared to 3-weeks regimen to induce ototoxicity.^[22]^ Therefore, a weekly cisplatin regimen is more feasible than a 3-week regimen during RT after induction chemotherapy.

In addition to cisplatin, RT also directly causes ear complication due to mechanical disruption of surrounding structures, such as in the eustachian tube of the middle ear, in patients with NPC. The pathogenesis of RT-induced normal tissue injury is associated with different mechanisms, including DNA damage repair, inflammation, cell death, and matrix remodeling.^[23]^

Based on our result, age was the single most important risk factor for both ear complication and renal toxicity. Elderly patients are vulnerable to inflammation and poor wound healing and thus more sensitive to the damage caused by radiation. The type of RT is also an important factor influencing the development of late complications. Compared with conventional RT, IMRT further enhances the efficacy of RT for NPC and delivers less radiation to the adjacent structures, such as the parotid glands and temporomandibular joints.^[24]^ The results from of this study showed that ear complications were significantly more common with conventional RT (60%) than 3D/IMRT (20%, *P* < .001) without any difference in CR rate or survival outcomes.

Another factor associated with RT-induced toxicity is the radiation dose. Until now, no optimal radiation dose has been established after induction chemotherapy. According to our results, survival outcome was similar in patients with radiation dose of 5400 cGy or higher, and as expected, ear complications occurred frequently with increasing radiation dose. From this finding, the optimal radiation dose after induction chemotherapy may be suggested as 5400 to 6600 cGy without any difference in treatment outcomes.

Our study provides important data for the long-term complications with risk factors and the suggestion for the optimal dose or radiation after TPF induction chemotherapy; however, there are several limitations. First, this study was conducted retrospectively and detailed examinations such as an audiometry were not performed in all patients. Thus, it should be considered especially in elderly patients to predict hearing impairment. Second, other late complications such as cranial neuropathy/temporal lobe necrosis and eye complications were not routinely evaluated. Therefore, these complications should be considered when monitoring patients treated with induction chemotherapy followed by CCRT in NPC and further study with large scale is needed.

## 5 Conclusions

TPF induction chemotherapy followed by CCRT is a feasible treatment modality for improving the treatment outcomes of patients with locally advanced NPC. However, close follow-up for long-term complications including of ear complication are necessary, especially elderly or patients treated with conventional RT. In the future, establishing optimal cisplatin and radiation dose to reduce toxicity while maintaining an antitumor effect during CCRT is necessary, at least for the patients who showed CR after induction chemotherapy.

## Author contributions

**Data curation:** Hyeon Jong Kim, Jun Eul Hwang, Ik Joo Chung, Dong Hoon Lee, Yoon Tae Mi, Joon Kyoo Lee, Sang Chun Lim, Jae Wook Chung.

**Resources:** Woo Kyun Bae.

**Writing – original draft:** Sanghee Cho, Hyun Jeong Shim.
